# Crystal structure of (+)-*N*-[(1*R*,5*S*,6*S*,9*S*)-5-hydroxy­methyl-3,3,9-trimethyl-8-oxo-2,4,7-trioxabi­cyclo­[4.3.0]nonan-9-yl]acetamide

**DOI:** 10.1107/S2056989016006800

**Published:** 2016-04-29

**Authors:** Takeshi Oishi, Shun Tsuzaki, Tomoya Sugai, Takaaki Sato, Noritaka Chida

**Affiliations:** a School of Medicine, Keio University, Hiyoshi 4-1-1, Kohoku-ku, Yokohama 223-8521, Japan; b Department of Applied Chemistry, Faculty of Science and Technology, Keio University, Hiyoshi 3-14-1, Kohoku-ku, Yokohama 223-8522, Japan

**Keywords:** crystal structure, bicyclic compound, 1,3-dioxane, oxolane, hydrogen bond, hy­droxy group

## Abstract

In the title compound, C_12_H_19_NO_6_, the six-membered 1,3-dioxane ring adopts a chair-like conformation. The seat of this chair, containing two O atoms, is essentially planar, with a maximum deviation of 0.0021 (12) Å. The five-membered oxolane ring *cis*-fused to the 1,3-dioxane ring adopts an envelope form. The bridgehead C atom at the flap, which is bonded to the tetra­substituted C atom of the oxolane ring, deviates from the mean plane of other ring atoms by 0.539 (4) Å. In the crystal, classical O—H⋯O and N—H⋯O hydrogen bonds link the mol­ecules into a sheet structure enclosing an *R*_4_^4^(24) graph-set motif. Weak inter­molecular C—H⋯O inter­actions support the sheet formation.

## Abstract 2

In the title compound, the 1,3-dioxane ring is in a chair-like conformation, while the fused oxolane ring adopts an envelope form. In the crystal, classical O—H⋯O and N—H⋯O hydrogen bonds link the mol­ecules into a sheet structure.

## Chemical context   

Sphingofungin F [systematic name: (2*S*,3*R*,4*R*,5*S*,*E*)-2-amino-3,4,5-trihy­droxy-2-methyl-14-oxoicos-6-enoic acid] was isolated from the fermentation broth of *Paecilomyces variotii* by Horn *et al.* (1992 ▸). It shows anti­fungal activity by inhibition of the serine palmitoyltransferase to suppress the early step of biosynthesis of the sphingosines (Zweerink *et al.*, 1992 ▸). The structure of sphingofungin F features a hydro­philic *α*,*α*-disubstituted *α*-amino acid moiety possessing four contiguous stereocenters, connected to a hydro­phobic carbon chain by *E*-olefin. The title compound, which is equivalent to the hydro­philic part with correct stereochemistry, was provided in the total synthesis of sphingofungin F (Tsuzaki *et al.*, 2015 ▸).
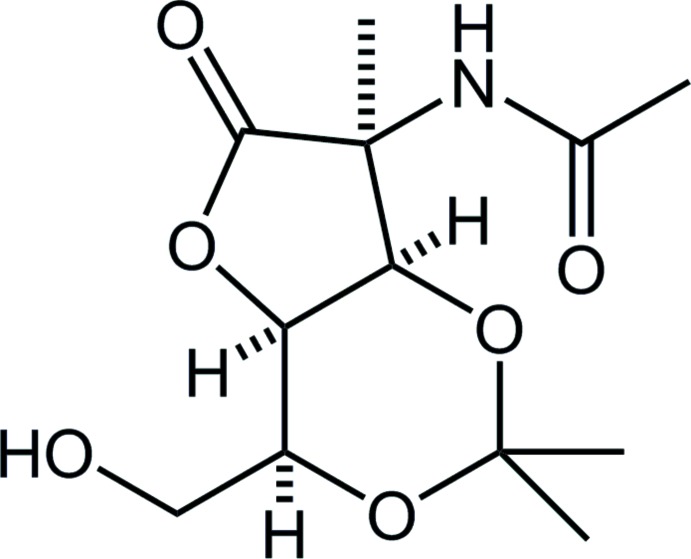


## Structural commentary   

**Figure 1:**
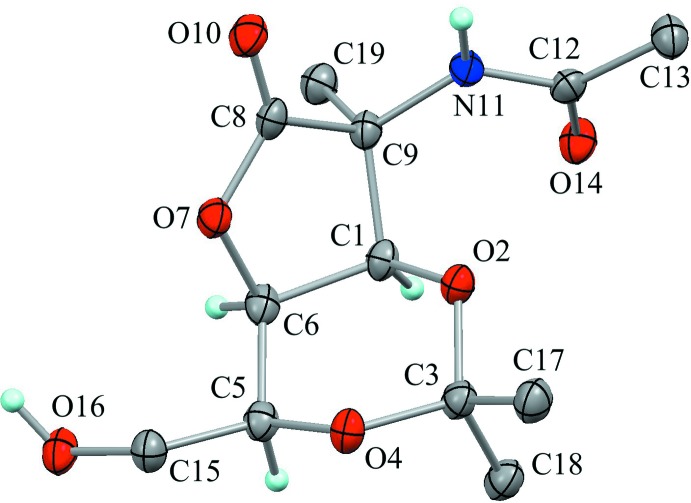
The mol­ecular structure of the title compound, showing the atom labeling. Displacement ellipsoids are drawn at the 50% probability level. Only H atoms connected to N, O and chiral C atoms are shown for clarity.

The mol­ecular structure of the title compound is shown in Fig. 1 ▸. The 1,3-dioxane ring (C1/O2/C3/O4/C5/C6) is in a chair-like conformation with puckering parameters of *Q* = 0.497 (3) Å, *θ* = 169.6 (3)°, *φ* = 116.8 (16)°, *Q*(2) = 0.090 (3) Å and *Q*(3) = −0.489 (3) Å. The seat of this chair (C1/O2/O4/C5) is essentially planar with a maximum deviation of 0.0021 (12) Å for O4, and atoms C6 and C3, positioned at the headrest and the footrest, respectively, deviate from the mean plane of the seat by 0.524 (4) and −0.646 (3) Å. The equatorially oriented C5—C15 and C3—C17 bonds make angles with the normal of the *Cremer & Pople plane* being 63.41 (18) and 63.35 (18)°, respectively, while the C1—C9 bond is a little tilted from the ideal equatorial position with an angle of 50.50 (17)° due to the ring-fusion system. The oxolane ring (C1/C6/O7/C8/C9), which is *cis*-fused to the 1,3-dioxane ring, adopts an envelope form with puckering parameters of *Q*(2) = 0.345 (3) Å and *φ*(2) = 254.7 (4)°. The bridgehead atom C1 deviates from the mean plane of the other four ring atoms by 0.539 (4) Å.

## Supra­molecular features   

**Figure 2:**
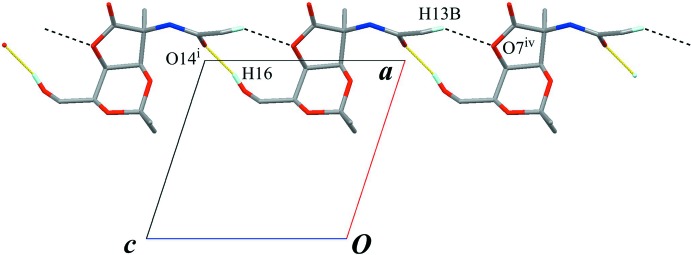
A partial packing diagram, viewed down the *b* axis, showing the chain structure running along the *c* axis. Yellow lines indicate the inter­molecular O—H⋯O hydrogen bonds. Black dashed lines indicate weak inter­molecular C—H⋯O inter­actions. Only H atoms involved in the hydrogen bonds are shown for clarity. [Symmetry codes: (i) *x*, *y*, *z* + 1; (iv) *x*, *y*, *z* − 1.]

**Figure 3:**
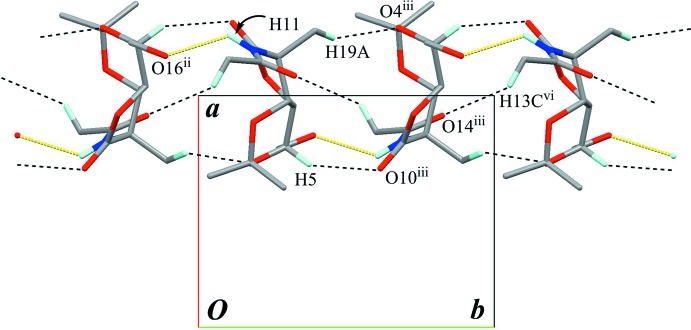
Another partial packing diagram, viewed down the *c* axis, showing the sheet structure parallel to (100). Yellow lines indicate the inter­molecular N—H⋯O hydrogen bonds. Black dashed lines indicate weak inter­molecular C—H⋯O inter­actions. Only H atoms involved in the hydrogen bonds are shown for clarity. [Symmetry codes: (ii) −*x* + 2, *y* − 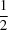, −*z* + 1; (iii) −*x* + 2, *y* + 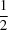, −*z* + 1; (vi) *x*, *y* + 1, *z* + 1.]

**Figure 4:**
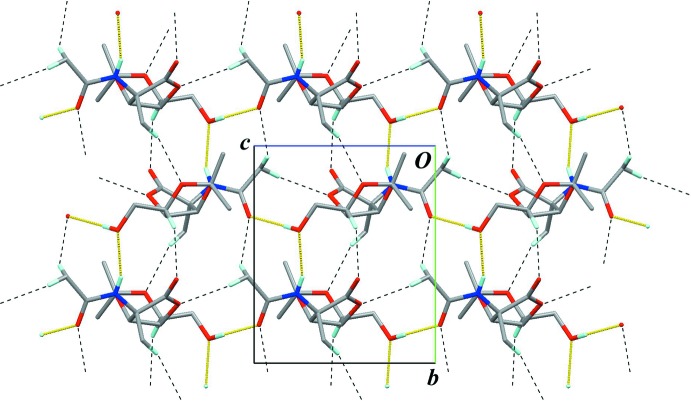
A packing diagram, viewed down the *a* axis, showing the hydrogen bonds in the sheet structure parallel to (100). Yellow lines indicate inter­molecular O—H⋯O and N—H⋯O hydrogen bonds. Black dashed lines indicate weak inter­molecular C—H⋯O inter­actions. Only H atoms involved in the hydrogen bonds are shown for clarity.

In the crystal, an O—H⋯O hydrogen bond (O16—H16⋯O14^i^; Table 1 ▸) connects the mol­ecules into a chain structure running along the *c* axis, with a *C*(10) graph-set motif (Fig. 2 ▸). A weak C—H⋯O inter­action (C13—H13*B*⋯O7^iv^; Table 1 ▸) supports formation of the chain. The chains are linked into a sheet structure parallel to (100) by an N—H⋯O hydrogen bond (N11—H11⋯O16^ii^; Table 1 ▸) which generates a *C*(8) graph-set motif (Fig. 3 ▸). Weak C—H⋯O inter­actions (C5—H5⋯O10^iii^, C19—H19*A*⋯O4^iii^ and C13—H13*C*⋯O14^v^; Table 1 ▸) are also observed between the chains. In this sheet structure, the classical O—H⋯O and N—H⋯O hydrogen bonds enclose an 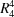
(24) graph-set motif, and the other weak C—H⋯O inter­actions add to the stability of the network (Fig. 4 ▸).

## Database survey   

**Figure 5:**
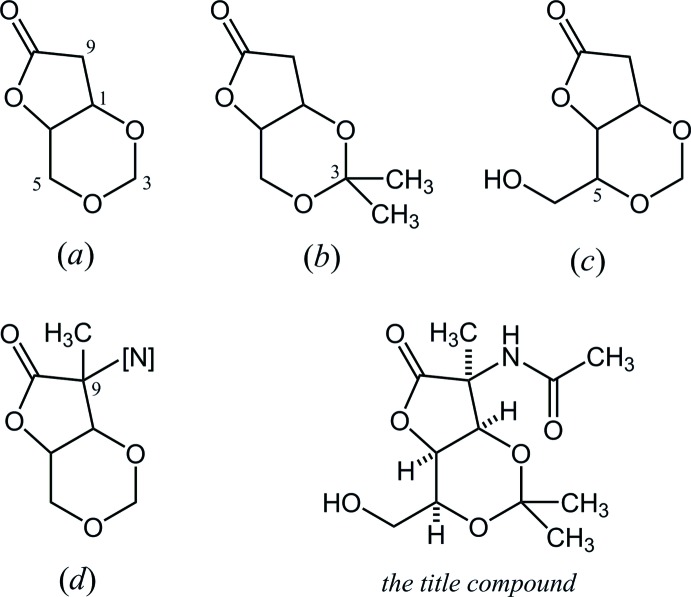
The core structures for database survey: (*a*) 2,4,7-trioxabi­cyclo­[4.3.0]nonan-8-one, and its derivatives with (*b*) 3,3-dimethyl, (*c*) 5-hy­droxy­methyl and (*d*) 9-methyl-9-*N*-substituents.

In the Cambridge Structural Database (CSD, Version 5.37, November 2015; Groom *et al.*, 2016 ▸), 18 structures containing a 2,4,7-trioxabi­cyclo­[4.3.0]nonan-8-one skeleton, (*a*), are registered (Fig. 5 ▸). These include five compounds (YISHIR and YISHUD: Han *et al.*, 1994 ▸; LAVVIO: Watkin *et al.*, 2005 ▸; ZINDEH and ZINDIL: Glawar *et al.*, 2013 ▸) with 3,3-dimethyl substituents, (*b*); one compound (NUIJAS: Henkel *et al.*, 1998 ▸) with 5-hy­droxy­methyl substituent, (*c*); and one compound (QIFFUH: Hotchkiss *et al.*, 2007 ▸) possessing a tetra­substituted carbon with nitro­gen at the C-9 position, (*d*). The conformations of the bicyclic systems in these seven structures are similar to those in the title compound: the 1,3-dioxane rings adopt chair-like forms, and the *cis*-fused oxolane rings adopt envelope forms with bridgehead C-1 position at the flap.

## Synthesis and crystallization   

The title compound was afforded in the total synthesis of sphingofungin F from a d-ribose derivative (Tsuzaki *et al.*, 2015 ▸). Purification was carried out by silica gel column chromatography, and colorless crystals were obtained from an ethyl acetate solution under a hexane-saturated atmosphere, by slow evaporation at ambient temperature. M.p. 497–498 K. [*α*]^28^_D_ + 157.7 (*c* 1.04, CHCl_3_). HRMS (ESI) *m*/*z* calculated for C_12_H_19_NO_6_Na^+^ [*M* + Na]^+^: 296.1110; found: 296.1104.

## Refinement   

Crystal data, data collection and structure refinement details are summarized in Table 2 ▸. C-bound H atoms were positioned geometrically with C—H = 0.95–1.00 Å, and constrained to ride on their parent atoms with *U*_iso_(H) = 1.2*U*_eq_(C) or 1.5*U*_eq_(methyl C). The hy­droxy H atom was placed guided by difference maps, with O—H = 0.84 Å and with *U*_iso_(H) = 1.5*U*_eq_(O). The amide H atom was also placed guided by difference maps, with N—H = 0.88 Å and with *U*_iso_(H) = 1.2*U*_eq_(N).

## Supplementary Material

Crystal structure: contains datablock(s) global, I. DOI: 10.1107/S2056989016006800/is5451sup1.cif

Structure factors: contains datablock(s) I. DOI: 10.1107/S2056989016006800/is5451Isup2.hkl

Supporting information file. DOI: 10.1107/S2056989016006800/is5451Isup3.cml

CCDC reference: 1475848

Additional supporting information:  crystallographic information; 3D view; checkCIF report

## Figures and Tables

**Table 1:** Hydrogen-bond geometry (Å, °)

*D*—H⋯*A*	*D*—H	H⋯*A*	*D*⋯*A*	*D*—H⋯*A*
O16—H16⋯O14^i^	0.84	1.91	2.742 (2)	168
N11—H11⋯O16^ii^	0.88	2.28	2.928 (3)	131
C5—H5⋯O10^iii^	1.00	2.42	3.289 (3)	145
C19—H19*A*⋯O4^iii^	0.98	2.52	3.386 (3)	147
C13—H13*B*⋯O7^iv^	0.98	2.55	3.433 (3)	150
C13—H13*C*⋯O14^v^	0.98	2.62	3.424 (3)	140

Symmetry codes: (i) 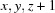; (ii) 
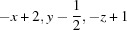
; (iii) 
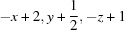
; (iv) 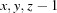; (v) 
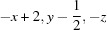
.

**Table 2:** Experimental details

Crystal data	
Chemical formula	C_12_H_19_NO_6_
*M*_r_	273.28
Crystal system, space group	Monoclinic, *P*2_1_
Temperature (K)	90
*a*, *b*, *c* (Å)	8.2102 (3), 9.9513 (3), 8.7480 (3)
β (°)	108.142 (2)
*V* (Å^3^)	679.20 (4)
*Z*	2
Radiation type	Cu *K*α
μ (mm^−1^)	0.91
Crystal size (mm)	0.14 × 0.14 × 0.07

Data collection	
Diffractometer	Bruker D8 Venture
Absorption correction	Multi-scan (*SADABS*; Bruker, 2014 ▸)
*T*_min_, *T*_max_	0.88, 0.94
No. of measured, independent and observed [*I* > 2σ(*I*)] reflections	8304, 2386, 2235
*R*_int_	0.039
(sin θ/λ)_max_ (Å^−1^)	0.596

Refinement	
*R*[*F* ^2^ > 2σ(*F* ^2^)], *wR*(*F* ^2^), *S*	0.032, 0.068, 1.00
No. of reflections	2386
No. of parameters	177
No. of restraints	1
H-atom treatment	H-atom parameters constrained
Δρ_max_, Δρ_min_ (e Å^−3^)	0.20, −0.18
Absolute structure	Flack *x* determined using 941 quotients [(*I* ^+^)−(*I* ^−^)]/[(*I* ^+^)+(*I* ^−^)] (Parsons *et al.*, 2013 ▸)
Absolute structure parameter	0.13 (11)

Computer programs: *APEX2* and *SAINT* (Bruker, 2014 ▸), *SHELXS2013* (Sheldrick, 2008 ▸), *SHELXL2014* (Sheldrick, 2015 ▸), *Mercury* (Macrae *et al.*, 2006 ▸), *publCIF* (Westrip, 2010 ▸) and *PLATON* (Spek, 2009 ▸).

## supplementary crystallographic information

### Crystal data

**Table:**

C_12_H_19_NO_6_	*D*_x_ = 1.336 Mg m^−^^3^
*M**_r_* = 273.28	Melting point = 497–498 K
Monoclinic, *P*2_1_	Cu *K*α radiation, λ = 1.54178 Å
*a* = 8.2102 (3) Å	Cell parameters from 5609 reflections
*b* = 9.9513 (3) Å	θ = 5.3–66.5°
*c* = 8.7480 (3) Å	µ = 0.91 mm^−^^1^
β = 108.142 (2)°	*T* = 90 K
*V* = 679.20 (4) Å^3^	Prism, colorless
*Z* = 2	0.14 × 0.14 × 0.07 mm
*F*(000) = 292	

### Data collection

**Table:**

Bruker D8 Venture diffractometer	2386 independent reflections
Radiation source: fine-focus sealed tube	2235 reflections with *I* > 2σ(*I*)
Multilayered confocal mirror monochromator	*R*_int_ = 0.039
Detector resolution: 10.4167 pixels mm^-1^	θ_max_ = 66.8°, θ_min_ = 5.3°
φ and ω scans	*h* = −9→9
Absorption correction: multi-scan (*SADABS*; Bruker, 2014)	*k* = −11→11
*T*_min_ = 0.88, *T*_max_ = 0.94	*l* = −10→10
8304 measured reflections	

### Refinement

**Table:**

Refinement on *F*^2^	Secondary atom site location: difference Fourier map
Least-squares matrix: full	Hydrogen site location: inferred from neighbouring sites
*R*[*F*^2^ > 2σ(*F*^2^)] = 0.032	H-atom parameters constrained
*wR*(*F*^2^) = 0.068	*w* = 1/[σ^2^(*F*_o_^2^) + 0.324*P*] where *P* = (*F*_o_^2^ + 2*F*_c_^2^)/3
*S* = 1.00	(Δ/σ)_max_ < 0.001
2386 reflections	Δρ_max_ = 0.20 e Å^−^^3^
177 parameters	Δρ_min_ = −0.18 e Å^−^^3^
1 restraint	Absolute structure: Flack *x* determined using 941 quotients [(*I*^+^)-(*I*^-^)]/[(*I*^+^)+(*I*^-^)] (Parsons *et al.*, 2013)
Primary atom site location: structure-invariant direct methods	Absolute structure parameter: 0.13 (11)

### Special details

**Table:**

Experimental. IR (KBr): 3311, 2967, 2896, 1785, 1658, 1539, 1170, 1117, 1057 cm^-1^; ^1^H NMR (500 MHz, CDCl_3_): *δ* (p.p.m.) 5.91 (s, 1H; H11), 4.82 (d, *J* = 2.0 Hz, 1H; H1), 4.37 (dd, *J* = 2.0, 1.7 Hz, 1H; H6), 4.21 (ddd, *J* = 7.2, 5.5, 1.7 Hz, 1H; H5), 3.92–3.79 (m, 2H; H15AB), 2.05 (bs, 1H; H16), 2.00 (s, 3H; H14ABC), 1.60 (s, 3H; H19ABC), 1.46 (s, 3H; H18ABC), 1.35 (s, 3H; H17ABC); ^13^C NMR (125 MHz, CDCl_3_): *δ* (p.p.m.) 176.9 (C), 170.1 (C), 98.8 (CH), 71.6 (CH), 71.5 (CH), 68.9 (CH), 62.3 (CH_2_), 61.6 (C), 29.1 (CH_3_), 23.5 (CH_3_), 19.2 (CH_3_), 18.0 (CH_3_).
Geometry. All esds (except the esd in the dihedral angle between two l.s. planes) are estimated using the full covariance matrix. The cell esds are taken into account individually in the estimation of esds in distances, angles and torsion angles; correlations between esds in cell parameters are only used when they are defined by crystal symmetry. An approximate (isotropic) treatment of cell esds is used for estimating esds involving l.s. planes.
Refinement. Refinement of *F*^2^ against ALL reflections. The weighted *R*-factor *wR* and goodness of fit *S* are based on *F*^2^, conventional *R*-factors *R* are based on *F*, with *F* set to zero for negative *F*^2^. The threshold expression of *F*^2^ > 2*σ*(*F*^2^) is used only for calculating *R*-factors(gt) *etc.* and is not relevant to the choice of reflections for refinement. *R*-factors based on *F*^2^ are statistically about twice as large as those based on *F*, and *R*-factors based on ALL data will be even larger.

### Fractional atomic coordinates and isotropic or equivalent isotropic displacement parameters (Å^2^)

**Table:**

	*x*	*y*	*z*	*U*_iso_*/*U*_eq_	
C1	0.9761 (3)	0.3025 (3)	0.3147 (3)	0.0188 (6)	
H1	0.9423	0.3845	0.2459	0.023*	
O2	0.8860 (2)	0.18536 (19)	0.2398 (2)	0.0201 (4)	
C3	0.7125 (3)	0.1762 (3)	0.2378 (3)	0.0206 (6)	
O4	0.7068 (2)	0.17674 (19)	0.3987 (2)	0.0210 (4)	
C5	0.7792 (3)	0.2941 (3)	0.4887 (3)	0.0204 (6)	
H5	0.7051	0.3729	0.4412	0.024*	
C6	0.9578 (3)	0.3214 (3)	0.4822 (3)	0.0199 (6)	
H6	0.9921	0.4151	0.5201	0.024*	
O7	1.0816 (2)	0.22653 (18)	0.5827 (2)	0.0209 (4)	
C8	1.2000 (3)	0.1928 (3)	0.5118 (3)	0.0197 (6)	
C9	1.1663 (3)	0.2685 (3)	0.3525 (3)	0.0188 (6)	
O10	1.3137 (2)	0.1152 (2)	0.5716 (2)	0.0250 (5)	
N11	1.2014 (3)	0.1803 (2)	0.2341 (2)	0.0189 (5)	
H11	1.2597	0.1056	0.2656	0.023*	
C12	1.1456 (3)	0.2118 (3)	0.0764 (3)	0.0199 (6)	
C13	1.1567 (4)	0.1008 (3)	−0.0361 (3)	0.0243 (6)	
H13A	1.2479	0.0382	0.0198	0.037*	
H13B	1.1822	0.139	−0.1294	0.037*	
H13C	1.0471	0.0528	−0.0722	0.037*	
O14	1.0868 (2)	0.32336 (19)	0.0272 (2)	0.0232 (4)	
C15	0.7705 (4)	0.2708 (3)	0.6581 (3)	0.0223 (6)	
H15A	0.6511	0.2505	0.6536	0.027*	
H15B	0.8432	0.1931	0.7074	0.027*	
O16	0.8286 (2)	0.3877 (2)	0.7522 (2)	0.0251 (4)	
H16	0.9172	0.3693	0.8284	0.038*	
C17	0.6531 (4)	0.0385 (3)	0.1716 (3)	0.0265 (6)	
H17A	0.735	−0.0292	0.2314	0.04*	
H17B	0.6455	0.035	0.0577	0.04*	
H17C	0.54	0.0202	0.1828	0.04*	
C18	0.6035 (4)	0.2878 (3)	0.1367 (3)	0.0248 (6)	
H18A	0.646	0.3752	0.1842	0.037*	
H18B	0.484	0.2765	0.1339	0.037*	
H18C	0.6104	0.2834	0.027	0.037*	
C19	1.2807 (3)	0.3934 (3)	0.3836 (3)	0.0225 (6)	
H19A	1.2492	0.4528	0.4593	0.034*	
H19B	1.2653	0.4411	0.2821	0.034*	
H19C	1.4009	0.3663	0.4295	0.034*	

### Atomic displacement parameters (Å^2^)

**Table:**

	*U*^11^	*U*^22^	*U*^33^	*U*^12^	*U*^13^	*U*^23^
C1	0.0219 (13)	0.0169 (14)	0.0158 (12)	−0.0014 (11)	0.0034 (10)	0.0009 (11)
O2	0.0200 (9)	0.0209 (10)	0.0178 (8)	−0.0027 (8)	0.0033 (7)	−0.0019 (8)
C3	0.0201 (13)	0.0240 (14)	0.0163 (12)	−0.0004 (12)	0.0036 (10)	0.0020 (13)
O4	0.0253 (10)	0.0196 (10)	0.0165 (9)	−0.0042 (8)	0.0044 (7)	−0.0005 (8)
C5	0.0235 (14)	0.0174 (14)	0.0190 (13)	0.0008 (11)	0.0049 (10)	−0.0018 (12)
C6	0.0247 (15)	0.0149 (14)	0.0179 (13)	0.0023 (11)	0.0035 (11)	−0.0008 (11)
O7	0.0227 (10)	0.0232 (11)	0.0155 (9)	0.0015 (8)	0.0038 (7)	0.0010 (8)
C8	0.0202 (13)	0.0199 (14)	0.0162 (12)	−0.0041 (11)	0.0016 (10)	−0.0031 (12)
C9	0.0217 (14)	0.0185 (14)	0.0150 (12)	0.0001 (10)	0.0039 (10)	−0.0021 (11)
O10	0.0265 (11)	0.0251 (11)	0.0199 (10)	0.0042 (9)	0.0021 (8)	0.0016 (9)
N11	0.0204 (11)	0.0182 (11)	0.0168 (11)	0.0034 (9)	0.0040 (8)	−0.0010 (10)
C12	0.0160 (12)	0.0230 (15)	0.0196 (13)	−0.0034 (11)	0.0043 (10)	−0.0029 (12)
C13	0.0298 (16)	0.0242 (15)	0.0185 (13)	0.0026 (12)	0.0069 (11)	−0.0003 (12)
O14	0.0292 (10)	0.0189 (11)	0.0190 (9)	0.0018 (8)	0.0040 (8)	0.0024 (8)
C15	0.0244 (15)	0.0226 (15)	0.0191 (13)	−0.0023 (11)	0.0057 (11)	−0.0022 (12)
O16	0.0310 (11)	0.0215 (10)	0.0188 (10)	0.0032 (9)	0.0018 (8)	−0.0035 (9)
C17	0.0260 (15)	0.0288 (16)	0.0223 (14)	−0.0040 (12)	0.0040 (12)	−0.0041 (13)
C18	0.0223 (14)	0.0299 (17)	0.0195 (13)	−0.0006 (12)	0.0028 (11)	0.0035 (13)
C19	0.0229 (14)	0.0216 (14)	0.0219 (14)	−0.0021 (11)	0.0053 (11)	−0.0020 (13)

### Geometric parameters (Å, º)

**Table:**

C1—O2	1.426 (3)	N11—H11	0.88
C1—C6	1.530 (4)	C12—O14	1.233 (3)
C1—C9	1.530 (4)	C12—C13	1.501 (4)
C1—H1	1.0	C13—H13A	0.98
O2—C3	1.422 (3)	C13—H13B	0.98
C3—O4	1.423 (3)	C13—H13C	0.98
C3—C17	1.509 (4)	C15—O16	1.419 (3)
C3—C18	1.525 (4)	C15—H15A	0.99
O4—C5	1.431 (3)	C15—H15B	0.99
C5—C6	1.510 (4)	O16—H16	0.84
C5—C15	1.523 (4)	C17—H17A	0.98
C5—H5	1.0	C17—H17B	0.98
C6—O7	1.463 (3)	C17—H17C	0.98
C6—H6	1.0	C18—H18A	0.98
O7—C8	1.349 (3)	C18—H18B	0.98
C8—O10	1.199 (3)	C18—H18C	0.98
C8—C9	1.532 (4)	C19—H19A	0.98
C9—N11	1.453 (3)	C19—H19B	0.98
C9—C19	1.530 (4)	C19—H19C	0.98
N11—C12	1.348 (3)		
			
O2—C1—C6	110.4 (2)	C12—N11—H11	119.7
O2—C1—C9	105.5 (2)	C9—N11—H11	119.7
C6—C1—C9	102.6 (2)	O14—C12—N11	122.6 (3)
O2—C1—H1	112.6	O14—C12—C13	122.0 (2)
C6—C1—H1	112.6	N11—C12—C13	115.4 (2)
C9—C1—H1	112.6	C12—C13—H13A	109.5
C3—O2—C1	115.6 (2)	C12—C13—H13B	109.5
O2—C3—O4	109.22 (18)	H13A—C13—H13B	109.5
O2—C3—C17	105.4 (2)	C12—C13—H13C	109.5
O4—C3—C17	106.1 (2)	H13A—C13—H13C	109.5
O2—C3—C18	111.4 (2)	H13B—C13—H13C	109.5
O4—C3—C18	112.1 (2)	O16—C15—C5	109.3 (2)
C17—C3—C18	112.2 (2)	O16—C15—H15A	109.8
C3—O4—C5	114.3 (2)	C5—C15—H15A	109.8
O4—C5—C6	111.6 (2)	O16—C15—H15B	109.8
O4—C5—C15	105.8 (2)	C5—C15—H15B	109.8
C6—C5—C15	113.9 (2)	H15A—C15—H15B	108.3
O4—C5—H5	108.5	C15—O16—H16	109.5
C6—C5—H5	108.5	C3—C17—H17A	109.5
C15—C5—H5	108.5	C3—C17—H17B	109.5
O7—C6—C5	111.2 (2)	H17A—C17—H17B	109.5
O7—C6—C1	103.9 (2)	C3—C17—H17C	109.5
C5—C6—C1	113.6 (2)	H17A—C17—H17C	109.5
O7—C6—H6	109.3	H17B—C17—H17C	109.5
C5—C6—H6	109.3	C3—C18—H18A	109.5
C1—C6—H6	109.3	C3—C18—H18B	109.5
C8—O7—C6	110.5 (2)	H18A—C18—H18B	109.5
O10—C8—O7	122.3 (2)	C3—C18—H18C	109.5
O10—C8—C9	127.5 (2)	H18A—C18—H18C	109.5
O7—C8—C9	110.1 (2)	H18B—C18—H18C	109.5
N11—C9—C19	111.8 (2)	C9—C19—H19A	109.5
N11—C9—C1	113.3 (2)	C9—C19—H19B	109.5
C19—C9—C1	112.8 (2)	H19A—C19—H19B	109.5
N11—C9—C8	109.3 (2)	C9—C19—H19C	109.5
C19—C9—C8	108.1 (2)	H19A—C19—H19C	109.5
C1—C9—C8	100.9 (2)	H19B—C19—H19C	109.5
C12—N11—C9	120.6 (2)		
			
C6—C1—O2—C3	51.4 (3)	C6—O7—C8—C9	1.5 (3)
C9—C1—O2—C3	161.5 (2)	O2—C1—C9—N11	33.6 (3)
C1—O2—C3—O4	−59.9 (3)	C6—C1—C9—N11	149.3 (2)
C1—O2—C3—C17	−173.5 (2)	O2—C1—C9—C19	161.8 (2)
C1—O2—C3—C18	64.6 (3)	C6—C1—C9—C19	−82.5 (3)
O2—C3—O4—C5	59.4 (3)	O2—C1—C9—C8	−83.1 (2)
C17—C3—O4—C5	172.6 (2)	C6—C1—C9—C8	32.6 (3)
C18—C3—O4—C5	−64.6 (3)	O10—C8—C9—N11	39.2 (4)
C3—O4—C5—C6	−52.2 (3)	O7—C8—C9—N11	−141.7 (2)
C3—O4—C5—C15	−176.5 (2)	O10—C8—C9—C19	−82.7 (3)
O4—C5—C6—O7	−74.1 (3)	O7—C8—C9—C19	96.4 (2)
C15—C5—C6—O7	45.7 (3)	O10—C8—C9—C1	158.7 (3)
O4—C5—C6—C1	42.7 (3)	O7—C8—C9—C1	−22.2 (3)
C15—C5—C6—C1	162.4 (2)	C19—C9—N11—C12	−75.4 (3)
O2—C1—C6—O7	79.1 (2)	C1—C9—N11—C12	53.3 (3)
C9—C1—C6—O7	−32.9 (2)	C8—C9—N11—C12	164.9 (2)
O2—C1—C6—C5	−41.8 (3)	C9—N11—C12—O14	11.3 (4)
C9—C1—C6—C5	−153.9 (2)	C9—N11—C12—C13	−167.6 (2)
C5—C6—O7—C8	142.6 (2)	O4—C5—C15—O16	−175.7 (2)
C1—C6—O7—C8	20.1 (3)	C6—C5—C15—O16	61.4 (3)
C6—O7—C8—O10	−179.4 (2)		

### Hydrogen-bond geometry (Å, º)

**Table:**

*D*—H···*A*	*D*—H	H···*A*	*D*···*A*	*D*—H···*A*
O16—H16···O14^i^	0.84	1.91	2.742 (2)	168
N11—H11···O16^ii^	0.88	2.28	2.928 (3)	131
C5—H5···O10^iii^	1.00	2.42	3.289 (3)	145
C19—H19*A*···O4^iii^	0.98	2.52	3.386 (3)	147
C13—H13*B*···O7^iv^	0.98	2.55	3.433 (3)	150
C13—H13*C*···O14^v^	0.98	2.62	3.424 (3)	140

Symmetry codes: (i) *x*, *y*, *z*+1; (ii) −*x*+2, *y*−1/2, −*z*+1; (iii) −*x*+2, *y*+1/2, −*z*+1; (iv) *x*, *y*, *z*−1; (v) −*x*+2, *y*−1/2, −*z*.
